# Ocular Tuberculosis with Multiple Cerebral Abscesses

**DOI:** 10.1155/2012/606741

**Published:** 2012-03-26

**Authors:** Saidin Nor-Masniwati, Embong Zunaina, Yaakub Azhany

**Affiliations:** Department of Ophthalmology, Universiti Sains Malaysia, Kubang Kerian, Kelantan 16150, Malaysia

## Abstract

A 23-year-old Malay man presented with headache for one-month duration. It was associated with painless blurring of vision of the right eye. He had loss of appetite and reduced weight but no night sweats or hemoptysis. His visual acuity on the right eye was 6/45 and improved to 6/15 with pinhole. Right fundus examination revealed a choroidal tuberculoma located at one disc diameter away from optic disc superiorly with mild vitritis. Systemic examinations revealed no significant finding. Mantoux test reading was 22 mm with erythrocyte sedimentation rate that was 14 mm/h. Other blood investigations were negative with normal chest radiography. The computerized tomography scan of the brain revealed multiple cerebral abscesses. A clinical diagnosis of right ocular tuberculosis with multiple cerebral abscesses was made. He was treated with antituberculosis chemotherapy for one year which divided into intensive phase for three months and maintenance phase for nine months. Cerebral abscesses resolved after three months of antituberculosis drugs and at one-year follow-up, and the choroidal tuberculoma resolved completely with scar formation and significant macular striae.

## 1. Introduction

Tuberculosis (TB) is an airborne infectious disease that is caused by *Mycobacterium tuberculosis* and related with formation of granulomatous infection that disseminated by haematogenous spread from the lungs. The extrapulmonary involvements were seen in cardiovascular, gastrointestinal, genitourinary, and central nervous system, skin, and eye which may or may not be associated with pulmonary tuberculosis [1]. The incidence of intraocular tuberculosis is rare and is most likely due to postprimary infection from the haematogenous spread [2]. 

We report a case of ocular tuberculosis with multiple cerebral abscesses in an immunocompetent young man.

## 2. Case Report

A 23-year-old Malay gentleman factory worker presented with generalised headache for one-month duration. It was associated with nausea and vomiting. He experienced painless progressive blurring of vision of the right eye two weeks later which he described as inferior visual field defect. He had loss of appetite and significant reduced weight but no night sweats or hemoptysis. Otherwise, there was no history of contact with pulmonary tuberculosis patient, drug abuse, blood transfusion, and high-risk behaviour. He has no other medical illness.

**Figure 1:**
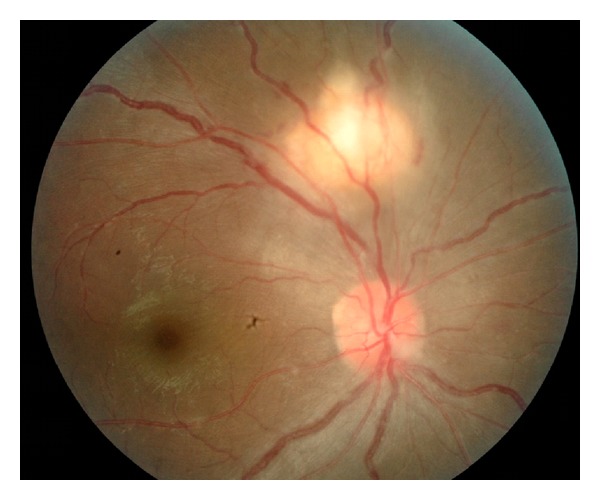
Right fundus showed a choroidal tuberculoma, measuring one and a half disc diameter located at one disc diameter away from optic disc superiorly.

His visual acuity on the right eye was 6/45 and improved to 6/15 with pinhole. There was no relative afferent pupillary defect. The right eye showed mild vitritis with no anterior chamber reaction. Right fundus examination revealed a choroidal raised lesion, yellowish in colour at the centre with pinkish fluffy edges, measuring one and a half disc diameter, and located at one disc diameter away from optic disc superiorly (Figure 1). The arcades vessels appeared tortuous and dilated with perivascular sheathing at supero- temporal and nasal arcade. The optic disc looked swollen and hyperaemic with gliosis at inferonasal disc margin. The left eye was normal.

Systemic examinations revealed no cervical, axillary, or inguinal lymphadenopathy. The respiratory, cardiovascular, and central nervous systems examination remained no abnormalities.

**Figure 2:**
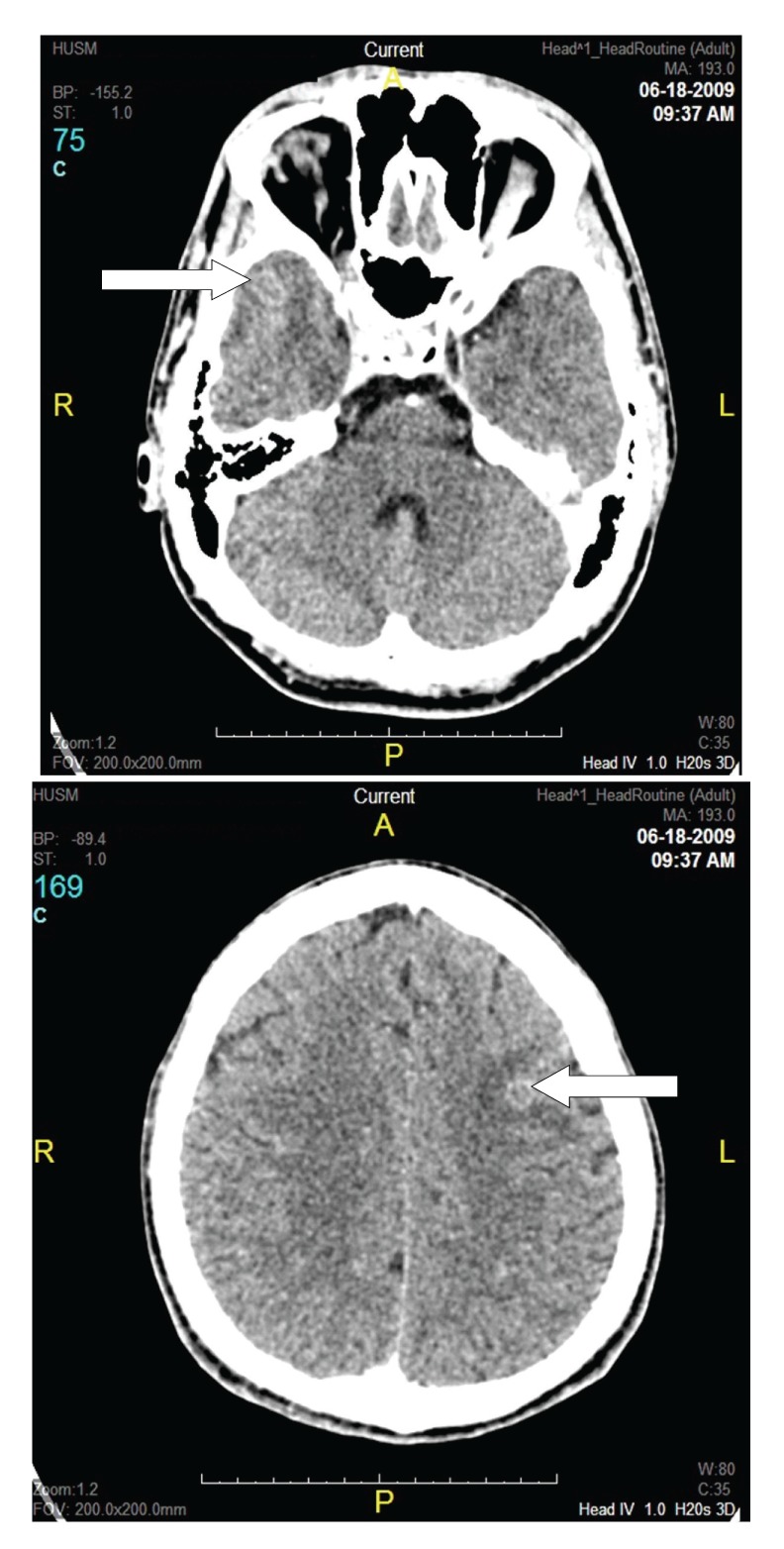
CT scan of brain and orbit showed ill-defined peripherally enhancing iso to hypodense lesions with perilesional edema at right frontal and left temporal lobe consistent with cerebral abscesses.

The ultrasonography of the right eye showed no evidence of retinal detachment, acoustic hollowing, or uveal excavation to suggest choroidal melanoma. The chest radiograph revealed no focal lesion or consolidation to suggest pulmonary tuberculosis. Mantoux test showed reading of 22 mm. Full blood count result showed that no significant finding with erythrocyte sedimentation rate (ESR) was 14 mm/h. Serum toxoplasmosis of IgM and IgG was negative. The syphilis and retroviral serology were nonreactive, and connective tissue results also inconclusive. The computerized tomography (CT) scan of the brain and orbit revealed multiple ill-defined peripherally enhancing iso to hypodense lesions with perilesional edema at right frontal and left temporal lobe consistent with cerebral abscesses (Figure 2).

A clinical diagnosis of right choroidal tuberculoma with multiple cerebral abscesses suggestive of tuberculosis infection was made. He was treated with antituberculosis chemotherapy for one year which divided into intensive phase for three months (tablet ethambutol 1200 mg daily, tablet pyrazinamide 1500 mg daily, tablet isoniazid 300 mg daily, tablet rifampicin 600 mg daily, and tablet vitamin B6 10 mg daily) and maintenance phase for nine months (tablet isoniazid 900 mg biweekly, tablet rifampicin 600 mg biweekly, and tablet vitamin B6 10 mg biweekly) by chest physician.

**Figure 3:**
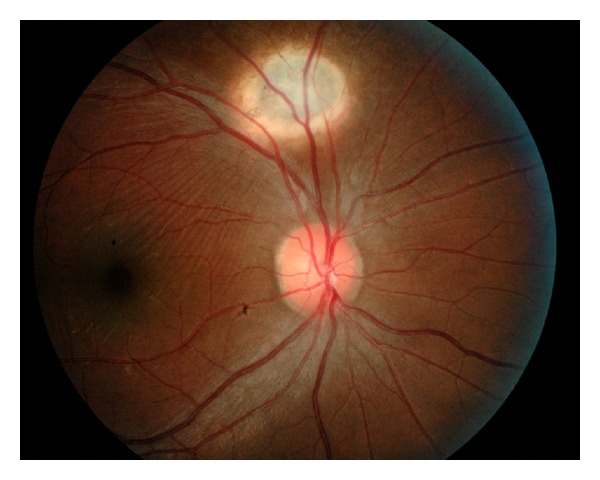
Right fundus showed the choroidal tuberculoma resolved completely with scar formation and significant macular striae.

On follow-up, the repeated CT scan of the brain after three months of antituberculosis drugs showed resolved cerebral abscesses. At the same time, his right visual acuity was improved to 6/6 and choroidal tuberculoma of the right fundus appeared to have well-defined margin with adjacent retinal striae. At one-year follow-up, the choroidal tuberculoma resolved completely with scar formation and significant macular striae (Figure 3).

## 3. Discussion

The clinical presentations in intraocular tuberculosis are anterior uveitis, intermediate uveitis, posterior or panuveitis, retinitis, vasculitis, neuroretinitis, optic neuropathy, endophthalmitis, and panophthalmitis [1]. Posterior segment is the most common clinical manifestation of intraocular involvement that affected choroid. Patient might presents with choroidal tubercles (small nodules), choroidal tuberculoma (size more than 4 mm), subretinal abscesses, and choroiditis [1]. The choroidal tubercles were the most common sign seen, and tuberculoma may be mimicking the ocular tumour since it may be appeared as solitary subretinal mass and surrounding exudative retinal detachment.

Occasionally, the patient may present mimicking or masquerading as ocular tumour [2, 3]. Demirci et al. had reviewed 5 cases ranging from 23 to 37 years old of ocular tuberculosis, 3 of them had choroidal tuberculoma with retinal detachment, one had panophthalmitis, and one had endopthalmitis [2]. Four cases were referred to oncology for suspecting choroidal melanoma and one patient suspected conjunctival melanoma. Our patient presented with choroidal tuberculoma, and B scan showed a feature of choroidal mass. Based on Mantoux test, he was diagnosed as ocular tuberculosis.

Central nervous system involvement occurs in 2–5% of all patients with tuberculosis [4]. Cesur et al. reported 2 cases of intracranial tuberculoma as a complication of tuberculosis meningitis which presented earlier as cavitary tuberculosis and subdural empyema [5]. Coexistent of ocular tuberculosis with intracranial manifestation of tuberculosis has been reported [6, 7]. Our patient presented earlier with headache and vomiting that showed sign of increase intracranial pressure and CT scan reported as frontotemporal lobe abscesses.

The patient should be referred to the physician with tuberculosis infection expert for opinion and comanage the case before initiating the treatment. The response of treatment should be assessed by the respective team to reach the therapeutic level. The regimens for ocular TB are similar for pulmonary or extrapulmonary tuberculosis. Gupta et al. revealed that 95% of 150 with presumed or confirmed tuberculosis that received treatment responded and showed resolution of inflammation [1]. The initial regime consisted of isoniazid, rifampicin, ethambutol, and pyrazinamide for 2 to 3 months. Only isoniazid and rifampicin were continued for 9 to 12 months [1]. Our patient received antituberculosis treatment from chest physician with intensive regime that was continued for three months and the maintenance treatment for nine months. He was monitored for the resolution of ocular and cerebral tuberculosis by neurology and eye team.

Multiple organ involvement needs multidisciplinary management in order to monitor patient's compliance, complication, and improvement of the condition.
